# Systemic Loxoscelism, Less Frequent but More Deadly: The Involvement of Phospholipases D in the Pathophysiology of Envenomation

**DOI:** 10.3390/toxins15010017

**Published:** 2022-12-27

**Authors:** Luiza Helena Gremski, Hanna Câmara da Justa, Nayanne Louise Costacurta Polli, Pedro Henrique de Caires Schluga, João Lucas Theodoro, Ana Carolina Martins Wille, Andrea Senff-Ribeiro, Silvio Sanches Veiga

**Affiliations:** 1 Department of Cell Biology, Federal University of Paraná (UFPR), Curitiba 81531-980, Brazil; 2 Department of Structural, Molecular Biology and Genetics, State University of Ponta Grossa (UEPG), Ponta Grossa 84030-900, Brazil

**Keywords:** brown spider, *Loxosceles*, venom, toxin, hemolysis, acute renal failure

## Abstract

Bites of *Loxosceles* spiders can lead to a set of clinical manifestations called loxoscelism, and are considered a public health problem in many regions. The signs and symptoms of loxoscelism are divided into cutaneous and systemic forms. The former is more frequent and includes signs of envenoming at the bite site or neighboring regions. Systemic loxoscelism, although much less frequent, is associated with complications, and can even lead to death. It may include intravascular hemolysis, acute renal failure, and thrombocytopenia. *Loxosceles* venoms are enriched with phospholipases D (PLDs), which are a family of isoforms found at intra-species and inter-species levels. Under experimental conditions, these enzymes reproduce the main clinical signs of loxoscelism, including an exacerbated inflammatory response at the bite site and dermonecrosis, as well as thrombocytopenia, intravascular hemolysis, and acute renal failure. The role of PLDs in cutaneous loxoscelism was described over forty years ago, when studies identified and purified toxins featured as sphingomyelinase D. More recently, the production of recombinant PLDs and discoveries about their structure and mechanism has enabled a deeper characterization of these enzymes. In this review, we describe these biochemical and functional features of *Loxosceles* PLDs that determine their involvement in systemic loxoscelism.

## 1. Introduction

*Loxosceles* spiders are cosmopolitan, with more than 140 different species described [1,2]. These spiders are involved in accidents with humans all over the world and are known by different names in distinct regions. In North America, they are known as recluse spiders, as they show a reclusive behavior, or as violin spiders, due to a design on their cephalothorax that resembles a violin. In South America, they are known as brown spiders, due to the characteristic brown coloring of their bodies [2,3,4,5]. The species with clinical significance that prevail in North America are *Loxosceles reclusa, L. arizonica*, and *L. deserta*. and in South America, *L. intermedia, L. laeta*, and *L. gaucho* [2,3,4,5].

*Loxosceles* venoms are colorless, crystalline, and have a high protein content. Unlike snakes that inject several milliliters of venom into victims, Brown spiders inoculate only a few microliters of venom [2,6,7]. The literature describes a few variations in the venoms of these arachnids, depending on species, gender, or physiological state of animals. When these venoms are submitted for analysis by two-dimensional electrophoresis, it is possible to observe fractions of proteins that prevail in the regions between 3–5 kDa (pI 7.5–10.0) and 20–40 kDa (pI 6.0–10.0) [2]. Among the toxins found in venoms, three families of toxins are greatly expressed, comprising about 95% of the transcripts coding for toxins in the venom glands: (i) Phospholipases D (PLD), also called dermonecrotic toxins (as they are mainly responsible for the typical skin lesions of loxoscelism); (ii) metalloproteases, characterized as astacins because of their similarity to the prototype protease identified in the lobster *Astacus astacus*; (iii) Knottins, or ICK (inhibitor cystine knot) peptides, with insecticidal activity. Other less expressed toxins are present, and include members of the serinoprotease families and serinoprotease inhibitors, characterized as serpins, hyaluronidases, allergen factors, and TCTP (translationally controlled tumor protein), a histamine-releasing factor [2,8,9,10]. 

**Figure 1:**
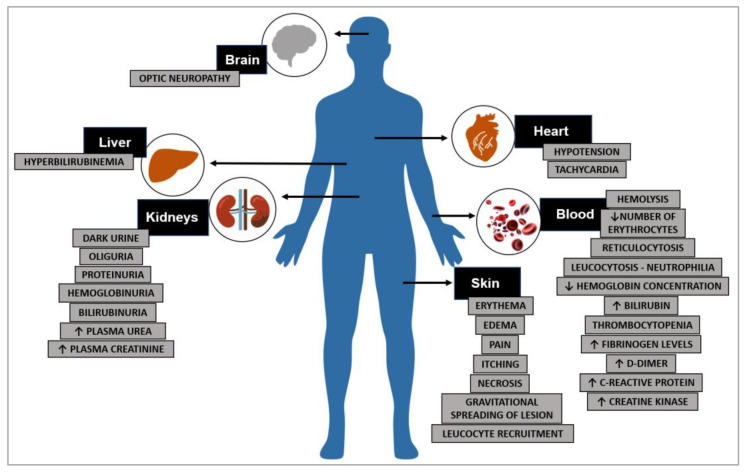
Tissues and organs affected by systemic and cutaneous loxoscelism, and main symptoms. Up arrows indicate increased values. Down arrows indicate reduced values.

The set of medical manifestations from bites by spiders of the *Loxosceles* genus is called loxoscelism, and is divided into two entities. Cutaneous loxoscelism, which prevails in accidents, and is reported in approximately 70% or more of victims. It is characterized by signs at the bite site or in nearby regions, including erythema, edema, pain, itching, and skin necrosis, as well as a gravitational spreading of the lesion, which is a hallmark sign of cutaneous loxoscelism [4,7,11]. On the other hand, systemic loxoscelism occurs less frequently than the cutaneous form, being reported in a range from about 1 to 27% of victims [4,11,12]. This wide variation depends on the *Loxosceles* species involved, the geographic region, and genetic characteristics of the victim. These systemic findings are responsible for the clinical complications seen in patients involved in spider accidents, and may include fever, intravascular hemolysis, hemolytic anemia, thrombocytopenia, and acute renal failure with hemoglobinuria, hematuria, and proteinuria (Figure 1) [2,4,5,7,11,13]. Under experimental conditions, liver and cardiac alterations have also been described [14,15].

The pathophysiological mechanism of cutaneous loxoscelism is well-understood, and many studies attribute the skin lesions at the bite site to the action of PLDs, which stimulate an unregulated and exacerbated inflammatory response that leads to a tissue damage (Figure 1) [7,8]. In turn, the mechanism of systemic loxoscelism appears to be more complex, as it affects different tissues, organs, and cells, such as the blood and kidneys. Data have pointed to the participation of PLDs as central agents in the systemic manifestations as well, playing a role in the pathophysiology of injuries [8,16,17,18]. However, some evidence indicates that other toxins may also contribute to systemic effects. Metalloproteases and hyaluronidases, for instance, may induce the spread of other venom toxins to neighboring tissues and possibly to systemic circulation, enabling the access of venom toxins to distant tissues [2,8,10]. In the following sections, we will describe the current physiological and molecular knowledge regarding the participation of loxosceles venom PLDs in systemic loxoscelism.

## 2. Intravascular Hemolysis and Hemolytic Anemia

Among the clinical signs described in systemic loxoscelism, intravascular hemolysis, followed by acute hemolytic anemia, represent the pathological changes that reflect the direct or indirect capacity of venom toxins to lyse human red blood cells inside blood vessels, releasing hemoglobin into the blood stream. This event is associated with all possible pathophysiological complications and contributes to nephrotoxic activity, which is one of the systemic manifestations. Mild forms of hemolytic reactions prevail after intravascular hemolysis due to a *Loxosceles* bite, and this is typically restricted to children and young people [19]. However, severe cases of hemolytic reactions have also been reported, but the reason remains unknown; it may be related to a genetic predisposition. 

The evidence of intravascular hemolysis in such victims comes from clinical data and laboratory findings, which have been reported in the scientific literature [2,5,20,21,22]. Laboratory analyses show alterations in the blood count, with a drop in the number of erythrocytes, decrease in hemoglobin concentration, reticulocytosis, jaundice, and changes in the urine, including hemoglobinuria, bilirubinuria, and proteinuria [2,5,21,22,23]. Several articles have described clinical cases after *Loxosceles* bites, reporting signs related to intravascular hemolysis and acute anemia in patients requiring hospitalization and blood transfusion. Of these, some publications have been cited with their main findings summarized in Table 1. In 1961, two cases of hemolytic anemia were first reported after accidents with unidentified venomous animals. The patients underwent blood transfusions, and in both cases, the authors discussed the involvement of *Loxosceles* spiders [24]. In addition, a retrospective study of clinical cases involving patients injured by *Loxosceles* spider bites in the United States reported the presence of acute intravascular hemolysis in victims. In this study, six African American children aged between 3 and 15 years old were bitten by *Loxosceles* spiders and admitted to the pediatric unit, presenting with fever, jaundice, and hemolytic anemia [25]. A 2-year retrospective study also reported six young injured patients with signs of hemolytic anemia who required hospital admission and, in some cases, blood transfusions [23]. Furthermore, in a retrospective study carried out in the United States for a period of 10 years, 26 cases of children bitten by *Loxosceles* spiders were analyzed. Among these patients, 50% had intravascular hemolysis and hemolytic anemia. These clinical manifestations showed bimodal behavior in their distribution over time, as some patients showed signs of early hemolytic anemia (about 2 days after the bite), and other patients presented hemolytic anemia approximately 7 days after the bite, which was considered late by the authors [26]. Another clinical case that confirmed the hemolytic activity of *Loxosceles* venom reported clinical data from a 9-year-old African American patient bitten by a spider identified as *L. reclusa*. After the third day of hospitalization, the patient presented with generalized exanthematous pustulosis and Coombs-positive hemolytic anemia. A decrease in hemoglobin levels was observed between the third and sixth day after admission, with elevated lactate dehydrogenase, transaminases, urobilinogen, and indirect bilirubin. The patient received blood transfusion and blood pressure maintenance support [27]. A thorough 20-year retrospective study (1995–2015) focusing on the hemolytic effect of brown spider venom was reported. In this study, 373 possible cases of patients injured with *Loxosceles* bites and treated at Vanderbilt University Medical Center (Nashville, TN, USA) were analyzed. A total of 115 patients showed signs of systemic loxoscelism, and among these, 58 were classified as mild cases and 57 as moderate or severe cases with hemolysis. In the group of patients with moderate to severe cases, 18 patients presented with a positive Coombs test, 9 were positive for C3 and IgG, 6 were positive only for IgG, and 3 were positive only for C3. These patients also showed a mean decrease in hemoglobin levels of 3.1 g/dL over an average of 2 days after admission. Total bilirubin and lactate dehydrogenase levels were twice the normal limits. Again, data showed a mean age of 14 years of age, ranging between 9 and 24 years old. Two patients died in this study [28]. Another retrospective study reported nine patients treated at the Health Science Center of the University of Tennessee (Memphis, TN, USA) with signs of systemic loxoscelism that manifested hemolytic anemia between 2 and 7 days after the accident, requiring blood transfusions and showing accumulation of microspherocytes in the blood count [29]. In addition, retrospective data from an 8-year period showed that among 97 patients bitten by *Loxosceles* spiders, 39 (40%) had hemolytic reactions, which appeared between days 1 and 7 after the accident. Some patients were DAT (direct antibody test) positive for IgG antibodies and 30 patients needed blood transfusions [19]. Recently, two case reports of systemic loxoscelism with hemolytic anemia were described. Based on the results of the DAT, both patients were diagnosed with warm autoimmune hemolytic anemia. Patients were treated with corticosteroids, received transfusions of packed red blood cells, and responded well to treatments [30]. 

Hemolytic anemia and intravascular hemolysis have been reported in accidents involving different *Loxosceles* species. For instance, some studies have described the involvement of *L. intermedia*, *L. laeta*, and *L. gaucho* from South America; species that prevail in North America, such as *L. reclusa*; and species from other locations, such as *L. similis* [2,5,22,23]. The fact that the clinical signs are very similar in accidents involving different species of *Loxosceles* denotes the idea that similar toxins are associated with the clinical manifestations and are conserved across different venoms. 

There have been some studies that discuss the indication of plasmapheresis in severe cases of intravascular hemolysis and hemolytic anemia triggered by *Loxosceles* bites. The first clinical case report described a *Loxosceles* accident of a 6-year-old boy, who presented with severe hemolysis, clinical of shock, hematological alterations, and renal failure. The patient underwent plasma exchange therapy, and after nine sessions of plasmapheresis, recovered from a 2 g/dL level of plasma hemoglobin to undetectable hemoglobin values [31]. Another publication reported a clinical case of a 17-year-old American patient bitten by a *Loxosceles* spider. The patient developed systemic loxoscelism with hemolytic anemia and severe intravascular hemolysis, which was refractory to a blood transfusion. Therapeutic plasma exchange was then performed, leading to recovery of the patient, who no longer needed transfusions. The authors discussed the application of this treatment modality in severe cases of intravascular hemolysis and refractory hemolytic anemia caused by accidents involving *Loxosceles* spiders [32]. Recently, a clinical case of a 49-year-old American patient bitten by a *Loxosceles reclusa* spider was reported. The victim presented severe signs of systemic loxoscelism complications, with intravascular hemolysis and hemolytic anemia that was refractory to multiple blood transfusions, and was submitted to hemodialysis. On hospital day 2, given the worsening of acute renal injury and continued hemolysis, the patient underwent treatment by plasmapheresis and showed signs of improvement in his hemoglobin levels 2 days after starting treatment. These authors also discussed the indication of plasmapheresis in severe cases of systemic loxoscelism with hemolysis and hemolytic anemia [33].

Currently, the mechanisms involved in the development of acute hemolytic anemia and intravascular hemolysis from *Loxosceles* bites are inconclusive. Likewise, it is not known why some patients are more prone to severe forms of intravascular hemolysis and hemolytic anemia than other victims, who do not present related clinical signs or only develop mild signs. The putative involvement of *Loxosceles* PLDs in these hemolytic events is supported by their in vitro hemolytic activities. It has been long known that venom components can induce in vitro hemolysis. For instance, the hemolytic and cytotoxic activities of *L. reclusa* crude venom was reported in studies from 1964 and 1965 to the present [34,35]. Forrester et al. [36] purified a toxin capable of degrading sphingomyelin and producing choline, thus being biochemically characterized as a sphingomyelinase D. This toxin caused in vitro lysis of human and sheep erythrocytes. This in vitro hemolytic activity induced by PLDs is preserved in toxins obtained from different species of *Loxosceles* spiders with wide geographic distributions, ranging from North America (*L. reclusa*) to South America (*L. intermedia*, *L. laeta*, and *L. gaucho*) [8,9,37,38]. This in vitro hemolytic activity has two distinct putative mechanisms. The first, named complement-dependent hemolysis, is induced by PLDs, but is dependent on the activation of the complement system, which indirectly promotes the lysis of red blood cells in vitro [39]. The second putative mechanism is called direct hemolysis because of the direct activity of PLDs on erythrocytes, which triggers hemolysis without the involvement of plasma proteins. This mechanism is dependent on the catalytic activity of these toxins, as wild-type toxins without catalytic activity or mutated recombinant molecules do not cause in vitro hemolysis [8,9,37,38,40]. 

Although lacking in mechanistic details, there are some known biochemical and cellular characteristics of the hemolytic action of PLDs. Human red blood cells exposed in vitro to *Loxosceles intermedia* recombinant PLDs went through morphological changes with the appearance of spherocytes, stomatocytes, and knizocytes, as well as cell size reduction [37]. Erythrocytes from distinct animals have different in vitro susceptibilities when treated with PLDs. Human, sheep, and rabbit erythrocytes were more sensitive to lysis than erythrocytes from horses, indicating an influence of the cell membrane lipid composition in this process [40]. This hypothesis was supported by the results of experiments carried out with artificial membranes treated with a *L. laeta* recombinant PLD, which showed that the action of the enzyme was influenced by the supramolecular organization and lipid composition of membranes [41]. Some studies showed that in vitro treatment of human red blood cells with purified PLDs caused activation of the alternative complement pathway, leading to complement-dependent hemolysis [39]. In addition, laboratory data on red blood cells collected from accidented patients with hemolysis and acute anemia showed positivity for the binding of complement factor C3 on these cells [23]. The degradation of phospholipids from the erythrocyte plasma membrane, such as sphingomyelin and lysophosphatidylcholine, and the influx of Ca^2+^ ions into the erythrocytes also appeared to be involved in the in vitro lysis mechanism [40]. 

In summary, there is clinical and laboratory evidence supporting hemolytic actions causing acute anemia in patients bitten by spiders of the *Loxosceles* genus. Data indicate the participation of members of the PLD family in the direct and indirect activities in vitro that induce morphological changes and lysis of human red blood cells. Although the mechanism of this hemolytic activity is not fully understood, these reported clinical events may be due to both direct and indirect activities, rendering erythrocytes more sensitive with the binding of phospholipases D, immunoglobulins, and proteins of complement systems, leading to lysis and clinical changes in patients. The hypothesis of antibody binding in phospholipases D-sensitized cells was supported by data showing that people who had never come into contact with these toxins, or had never been bitten by these spiders, naturally had antibodies that recognized these molecules [42].

## 3. Acute Renal Failure in Systemic Loxoscelism

Another reported manifestation of systemic loxoscelism is acute renal failure [7,11], which appears to be the leading cause of death in injured patients [2,4,5,22]. The impairment in renal functions of *Loxosceles* bite victims have been described confirmed with clinical findings, including an increase in blood creatinine and urea, and changes in the formation of urine, which becomes dark with reduced volume (oliguria), proteinuria, hematuria, and hemoglobinuria [2,4,5,7,22]. 

Cases of systemic loxoscelism with acute renal failure have been reported in patients of all ages, but appear to be more frequent in children; therefore, they require greater attention from clinicians [2,4,5,22,43]. Several case reports can be cited; the main findings of some representative studies are summarized in Table 2. For instance, there was a study describing two envenomation cases from Brazil, with patients with clinical signs of systemic loxoscelism, i.e., rhabdomyolysis and renal acute failure. One of the patients required dialysis for 50 days. Both patients presented with edema, erythema, and dermonecrosis at the bite site. Laboratory findings showed increased creatine kinase levels in both patients. The authors hypothesized that the rhabdomyolysis that resulted from intense tissue destruction caused by the venom may have contributed to acute renal failure. Thus, they suggested monitoring creatine kinase levels in patients bitten by spiders to alert clinicians of possible renal impairments or acute renal failure [44]. A retrospective analysis of six victims aged between 3 and 15 years old was reported with severe hemolytic anemia in all patients, and also presented with hyperkalemia and hypotension, with one child developing severe acute renal failure and requiring hemodialysis [25]. Another retrospective study conducted over a 10-year period with 26 American children bitten by *Loxosceles* spiders showed that 85% presented with necrotic skin lesions, 50% developed hemolytic anemia post-envenoming, 27% had rhabdomyolysis, and 12% manifested signs of acute renal failure [26]. There was also a detailed study of a 20-year-old American patient who was injured by a *Loxosceles* bite that showed clinical signs of renal function impairment and laboratory abnormalities including increased blood creatinine and urea; dark-red colored urine, with proteinuria, hematuria, and pyuria; positive Coombs; and hemolytic anemia. A renal biopsy showed disintegrating glomeruli, damage of the tubules, deposition of C3 in the mesangium and capillary loops, mild podocyte effacement, and mild endothelial edema. Based on the data compiled from renal biopsies and the patient’s clinical assessments, the authors affirmed that the main renal lesion was acute tubular necrosis, probably caused by the venom. As biopsy findings included glomeruli destruction, the authors suggested that the venom acted on glomerular permeability, leading to proteinuria [45]. Another study of a 50-year-old female patient bitten by a *Loxosceles* spider in India was reported. This patient developed dermonecrosis in one of her fingers, presenting with malaise, change in the color of urine, and oliguria a few hours after the accident. Increased blood urea (159 mg/dL) and creatine (6.6 mg/dL) were detected 48 h after the accident. The patient underwent hemodialysis, but after about 1 week of hospitalization, hemoglobin levels were still 4.7 g/dL and hemoglobinuria was present (though without hematuria). Other laboratory abnormalities included increased blood bilirubin, creatine kinase, lactate dehydrogenase, and reticulocytosis. A kidney biopsy showed acute tubular damage, pigment casts in some tubules, and interstitial edema, with normal glomeruli and vessels [46]. In a study carried out in Brazil between 2010 and 2015, specifically in an endemic region for *Loxosceles amazonica*, 45 patients were evaluated. Among them, 95.6% presented signs of cutaneous loxoscelism with dermonecrosis at the bite site and neighboring regions; 13.3% had acute renal failure with alterations in biochemical laboratory parameters and markers of renal function; and two patients died [47]. Finally, a retrospective study carried out in an endemic region of *Loxosceles reclusa* in the United States reported nine cases. Three of these patients had acute renal failure and all patients presented with intravascular hemolysis. The authors suggested that the acute renal failure had no correlation with the severity of hemolysis and reported that they were resolved with administration of fluids and steroids [29]. However, some studies associated the acute renal failure described in systemic loxoscelism with complications provoked by severe intravascular hemolysis, which, as previously discussed, is also a systemic manifestation of loxoscelism [22].

In addition, some authors associate acute renal failure with complications of cutaneous loxoscelism, mainly dermonecrosis and rhabdomyolysis. However, these manifestations do not show a causal relationship when studied under laboratory conditions. Some experimental animal models of loxoscelism, specifically in rats and mice, did not develop dermonecrosis when exposed to the crude venom or purified recombinant toxins. However, they presented altered renal function parameters, such as high blood urea level and changes in urine, such as proteinuria, hematuria, and hemoglobinuria [16,18,48]. In addition, mice were susceptible to the action of crude venom or purified toxins and died a few days after injections [18,48,49]. Moreover, rats exposed to the venom of *Loxosceles gaucho*, a common species in South America, under experimental conditions showed a significant decrease in the glomerular filtration rate, renal blood flow, and urine output, and augmented renal vascular resistance. Kidney biopsies showed acute degenerative alterations of tubular epithelial cells and the presence of cellular debris in the tubular lumen, but no glomerular or vascular alterations. Histological analyses showed deposition of myoglobin and hemoglobin in the kidneys [50]. 

The mechanisms by which *Loxosceles* venoms cause acute renal failure is not well understood. However, much knowledge has been gathered in this field by using recombinant isoforms of phospholipases D from *Loxosceles* venoms. They are the main components of *Loxosceles* venom, and their biological activity has been associated with post-envenoming renal lesions [16]. Under experimental conditions, PLDs reproduced key signs of kidney damage after exposure, such as changes in urine formation with proteinuria, hemoglobinuria, and hematuria. PLD exposition also induced histological changes in the kidney, such as glomerular edema and tubular necrosis with deposition of proteinaceous material inside the tubular lumen, but without signs of an inflammatory response in the renal structures [16,18], except causing animal death [18,49]. PLDs also led to cytotoxicity in kidney cell lines, such as MDCK (Madin-Darby canine kidney epithelial cells), acted as planted antigens in renal structures [16], and were eliminated in the urine of mice experimentally exposed to a recombinant PLD [18]. These data indicate the participation of these toxins in renal disturbances and failure. Furthermore, the catalytic activity of PLDs has been shown to be necessary to the nephrotoxicity of these toxins. This was shown by studies that used recombinant PLDs obtained by site-direct mutation techniques, with changes in amino acid residues of the catalytic site or other regions that modulate the biochemical activities of these enzymes. Results showed that these mutated PLDs were devoid of nephrotoxic or cytotoxic effects [9,16,18]. The participation of PLDs in post-envenoming renal failure caused by *Loxosceles* bites was reinforced by a study that showed that exposure of mice to a purified PLD caused acute renal failure, and the same treatment of human kidney cells (HK-2) in culture was cytotoxic, presenting in cell death [51]. As mentioned above, the death of loxoscelism victims is generally a consequence of acute renal failure complications. Recently, it was shown that mice previously immunized with inactive recombinant isoforms of PLDs (that conserved the molecular structure of wild-type PLDs) presented a protective immune response against the lethal activities of the crude venoms of *Loxosceles intermedia*, *L. laeta*, and *L. gaucho*, strengthening the evidence for the participation of PLDs in kidney damage after *Loxosceles* envenoming [49]. 

## 4. Hemostatic and Coagulatory Alterations in Systemic Loxoscelism

Besides the hematological disturbances that may develop in systemic loxoscelism, hemostatic and coagulatory alterations have also been described [2,4,5,7,22,43]. Coagulopathy and thrombocytopenia are rare manifestations and have no diagnostic criteria for disseminated intravascular coagulation [4]. Our understanding of these other hematological alterations, i.e., mild thrombocytopenia and changes in clotting times, along with hemolytic anemia, is based on reports of clinical cases and animal model studies exposed to crude venom and/or purified toxins. To highlight such alterations, some clinical reports of patients bitten by *Loxosceles* spiders have been described for endemic regions, and the main findings are summarized in Table 3. A retrospective study was carried out between the years of 2004–2006 on 81 patients bitten by *Loxosceles* spiders and admitted to Hospital Vital Brazil (Butantan Institute, São Paulo, Brazil) in an endemic region for *Loxosceles gaucho.* All data were retrieved between 1 and 9 days after accidents. Among the patients studied, 50.8% presented leukocytosis in the first week of hospital admission and 48.6% after second week. Signs of neutrophilia were observed in 37.8% of patients in the first week after admission, and 62.8% after the second week. Neutrophilic leukocytosis was the most frequent blood disorder detected. Levels of C reactive protein were increased in 85.3% of patients after 1 week and 41.2% of patients after 2 weeks. Fibrinogen concentration increased in 34% of patients within one week and 90.9% of patients after two weeks of admission. Thrombocytopenia was detected in 17.6% of patients after one week and 2.8% of patients after 2 weeks of admission. The international normalized ratio values were higher than 1.3 in 7.5% of patients, and lower than 1.0 in 13.2% of patients after hospitalization for one week. After one week of admission, 21.6% of patients presented PTT levels above 1.25. The D-dimer levels were increased in 53.5% of patients admitted after one week and 25% of patients after two weeks. According to the authors, none of the patients had a diagnosis of DIC (disseminated intravascular coagulation) [22]. One case report described an accident involving a 3-year-old child bitten by a *Loxosceles rufescens* specimen in an endemic region. The patient presented signs of hematological alterations with petechiae and erythema in the right big toe, probably the bite site. The victim also had thrombocytopenia, but did not present DIC. The thrombocytopenia was described as severe, with the presence of antibodies that recognized platelets in the plasma. The authors suggested an immune system-dependent thrombocytopenia-inducing mechanism, which was supported by the fact that the administration of corticosteroids triggered a rapid recovery in the number of blood platelets [52].

Under experimental conditions, the activity of crude venoms and purified PLDs on hematopoietic cells is well-established, indicating direct or indirect actions in vitro and in vivo. The infiltration of peripheral blood leukocytes into the dermis and adjacent structures in the bite site (characterizing a deep inflammatory response) has been shown using skin biopsies of rabbits exposed to venoms or purified PLDs from different *Loxosceles* species [8,9,20,53,54,55]. Migratory activity of peripheral blood leukocytes was observed, particularly segmented neutrophils that migrated to the dermis and interface of skeletal musculature with the hypodermis. This response reflects indirect endothelium-dependent leukocyte activation by PLDs [56] as in vitro data have described direct inhibitory action of venom on leukocytes [57]. For more details regarding the mechanisms of PLDs involved in the inflammatory response and necrosis, see Gremski et al. [8]. The direct action of venom components on platelets, particularly PLDs (native or recombinant), have been described in vitro, revealing platelet aggregation-promoting activities [58,59,60,61,62,63,64]. In addition, a detailed analysis of peripheral and bone marrow cells was performed using rabbits experimentally exposed to *Loxosceles intermedia* venom. The authors showed that the animals did not present significant red cell changes. However, there was a correlation between megakaryocyte depletion in bone marrow and peripheral thrombocytopenia, and between the decrease in the number of medullary neutrophils and peripheral leukocytosis. The authors concluded that thrombocytopenia and neutropenia in peripheral blood were likely due to marrow depletion, which could also be related to platelet and neutrophil migration to the inflammatory site. If the venom had an effect on the bone marrow, this effect was suggested to be transient, due to the recovery of cellularity after ending exposure to the venom [65]. Rabbits exposed to the venom of *Loxosceles gaucho* had thrombocytopenia during the first 48 h post-exposure, corroborating the venom activity on platelets [66]. The production of recombinant PLDs improved understanding of the involvement of PLDs both in leukocyte migration from peripheral blood to the skin in the bite site and in platelet aggregation in vitro. Several recombinant isoforms of PLDs from venoms of different *Loxosceles* species were able to stimulate in vitro platelet aggregation. For instance, at least four *L. intermedia* and one *L. gaucho* recombinant PLDs stimulated in vitro platelet aggregation [60,61,62,64] and exacerbated the inflammatory response, with massive leukocyte infiltration into the dermis of exposed animals [16,60,61,62]. The in vitro platelet aggregation induced by PLDs indicated a direct or indirect action on these cell fragments, suggesting involvement of these toxins in the thrombocytopenia observed in vivo and indicating a correlation with the cases of thrombocytopenia described in injured patients. Based on clinical observations of patients bitten by *Loxosceles* spiders, particularly those who developed systemic loxoscelism, the presence of disseminated intravascular coagulation was not reported; thus, it is considered a rare clinical manifestation [4,5,22]. Even so, as histopathological analysis of skin samples from animals injected with crude venoms or purified recombinant PLDs have shown thrombus formation inside blood vessels and *Loxosceles* venoms and PLDs have activated in vitro platelet aggregation [54,60,61,62], clinicians should be aware of clotting alterations that may appear after envenoming.

## 5. Liver, Brain, and Cardiac Disturbances after *Loxosceles* Bites

Although they are rare, some clinical case studies have reported cardiac, cerebral, and hepatic alterations in addition to the hematological and kidney disorders reported in patients bitten by *Loxosceles* spiders. 

For instance, a 63-year-old victim of a *Loxosceles* spider bite presented signs of optic neuropathy, characterized by a left relative afferent papillary defect, bilateral optic nerve pallor, decreased foveal sensitivity in the left eye, and bilateral visual compound defects [67]. A retrospective study was carried out in the United States with nine patients aged between 18 and 53 years-old who were bitten by *Loxosceles* spiders and developed moderate to severe systemic loxoscelism. Data showed that two patients manifested hemolytic anemia and hyperbilirubinemia, seven had tachycardia, and six presented with hypotension. Throughout the discussion of these clinical cases, emphasis was given to complications resulting from hemolytic effects [29]. Although hepatic lesions due to *Loxosceles* spider accidents are infrequent, Christoff et al. [14] showed that, under experimental conditions, rats exposed to crude venom or a recombinant PLD from *L. intermedia* presented liver function impairment. Signs of hepatotoxicity were evidenced by histological changes, such as tumefacted and apoptotic hepatocytes and leukocyte infiltration in the portal region, as well as steatosis 12 h after venom and PLD exposure. After 6 h of exposure to crude venom, hepatic markers such as alanine aminotransferase, aspartate aminotransferase, and gamma-glutamyl-transferase were also significantly altered. Animals exposed to purified PLD had milder symptoms. The authors concluded that the crude venom presented hepatotoxic activity and that the PLD toxin was partially responsible for this toxicity [14]. 

Although rare, patients bitten by *Loxosceles* spiders can develop heart complications. A clinical case was reported describing cardiac alterations in a 38-year-old Mexican patient envenomed by a spider that presumably belonged to the *Loxosceles* genus due to the characteristic necrotic condition at the bite site. The patient developed systemic loxoscelism and presented with systemic lupus erythematosus, exertional dyspnea, lower limb edema, tachycardia, and diastolic heart failure [68]. Recently, a clinical case of a 16-year-old male patient bitten by a *Loxosceles* spider was reported and the accident was confirmed by an enzyme immunoassay using a sample collected from the bite site. The patient presented with a decrease in blood pressure and cardiac disorders throughout the development of systemic loxoscelism. Biochemical parameters showed an increase in LDH, bilirubin, and plasma hemoglobin levels, consistent with intravascular hemolysis. Cardiac disorders were confirmed by the development of tachycardia and T-wave changes on the electrocardiogram, suggesting myocarditis. Blood markers for cardiac disorders, such as B-type natriuretic peptide, troponin-I, and creatine kinase, were increased. Finally, cardiac magnetic resonance confirmed myocarditis. The patient also showed pulmonary edema [69]. Table 4 summarizes the findings described in these clinical reports of systemic loxoscelism with neurological and cardiac involvement. 

The cardiotoxic activity of toxins from *Loxosceles* spider venoms was strengthened by laboratory findings from animals exposed to crude venom or purified toxins. An experimental study in which mice were exposed to crude venom and a recombinant PLD (rLiD1) from *L. intermedia* showed that both had cardiotoxic potential, leading to tissue changes in the heart of treated animals. Data showed that rLiD1 was detected in the heart and liver of exposed animals, which showed increased levels of creatine kinase in sera. Moreover, in vitro studies of the heart treated with recombinant PLD indicated impairments of cardiac functions. Cardiomyocytes isolated from animals exposed to recombinant PLD showed a significant increase in L-type calcium current density and intracellular Ca^2+^ transients [15]. Although reports of hepatotoxicity and cardiotoxicity in patients bitten by *Loxosceles* spiders are rare, clinicians who manage these patients should be aware of the physiological signs and markers of hepatic and cardiac functions, as crude venoms and purified recombinant PLDs under laboratory conditions have presented hepatotoxic and cardiotoxic effects.

## 6. Final Considerations

A remaining open question in systemic loxoscelism is why only 20–25% of injured patients develop signs of systemic intoxication and the other 75–80% have their injuries restricted to the bite site. Although different *Loxosceles* spider venoms show qualitatively conserved main components of toxins [2,8,10], the different distributional patterns of species involved in accidents and variant strains within the same species around the world help to explain these statistics [2,5,7]. Another explanation for this discrepancy may be the quantity of toxins injected at the time of the bite. Transcriptome analysis of the venom-producing glands from different species, such as *Loxosceles laeta*, *L. intermedia,* and *L. similis*, showed different proportions of transcripts coding for the PLD family of toxins, which, as described above, are the main toxins associated with the severity of accidents [70,71,72,73]. 

PLDs constitute both intra- and inter-species family of toxins [8]. In addition, different isoforms of PLDs may present significant variation regarding functional and enzymatic activities. Some isoforms are even devoid of enzymatic activities, presenting only residual biological activities [2,8]. Venoms enriched in PLD isoforms with low enzymatic and biological activities could explain lower toxicities in injured patients. The presence of certain comorbidities, such as diabetes, obesity, and allergies to other venom toxins, may be another explanation, as signs of loxoscelism are worse in these patients [7,74]. In addition, the age of victims seems to have an influence, as several studies have indicated greater severity of bites by *Loxosceles* in children, teenagers, and elderly victims [5,23,25]. The participation of genetic features that may increase the susceptibility of patients to the systemic effects of loxoscelism cannot be ruled out. So far, there has been no report of cellular or tissue markers associated with a predisposition to develop more severe manifestations of loxoscelism. The discovery of such markers will not only improve treatment protocols for systemic loxoscelism, but also move it toward new protection measures and improved differential diagnosis of victims. 

Another challenging aspect in systemic loxoscelism is that, currently, there are no efficient and reliable commercial laboratory methods for the diagnosis of systemic loxoscelism. As clinical features similar to loxoscelism can lead to misdiagnosis, a specific diagnostic test is necessary. Although *Loxosceles* spider bites have been associated with necrotic skin and systemic conditions for approximately 60 years [34,35,75,76], diagnoses are still made based on the spider being captured after the accident. Alternatively, a diagnosis can be based on the epidemiological profile of the region of the accident, as there are countries in South America, such as Brazil, Argentina, Chile, and Peru, in addition to the Southern states of the United States, where loxoscelism is frequent and even a public health problem. Finally, clinical signs and symptoms compatible with loxoscelism, such as skin necrosis at the bite site, gravitational spread of the lesion surrounding of the bite site, intravascular hemolysis, hemolytic anemia, and acute renal failure, can also be used in the diagnosis [2,5,8]. Experimental attempts at diagnosing loxoscelism using various invasive and non-invasive methods are reviewed in Gremski et al. [8]. More recently, a laboratory protocol was proposed to detect preclinical hemolysis in patients bitten by a *Loxosceles* spider. In a retrospective analysis of 275 patients injured by *Loxosceles* spider bites, 64 patients were identified with hemolytic anemia. Data showed that the combination of increased blood levels of total bilirubin and lactate dehydrogenase had high sensitivity and specificity in detecting patients developing hemolysis before a significant drop in hematocrit. Thus, the authors suggest that a simultaneous increase in the levels of these laboratory parameters is an effective marker to detect preclinical hemolysis in injured patients [77]. However, the description of a non-invasive, safe, and reproducible laboratory method with a good clinical correlation with accidents caused by *Loxosceles* spiders remains to be a challenge and a direction for future research.

In conclusion, as the scientific community is already providing various isoforms of recombinant PLDs, the application of these molecules as biological tools is a real possibility. The first isoforms of recombinant PLDs from *Loxosceles* spider venoms were obtained almost 20 years ago [78,79,80], and currently, there are dozens of these recombinant molecules from venoms of different species, either in their native (wild-type) forms or with incorporated mutations. As discussed above, the involvement of PLDs in the pathophysiological events of systemic loxoscelism enables these toxins to be major molecular targets for the development of specific treatments and laboratory diagnostic tests for *Loxosceles* spider bites. Production of a second-generation anti-*Loxosceles* sera based on mutated recombinant PLDs or synthetic chimera with epitopes of PLDs has become a real possibility in the toxinology field [8,9,38,49,81]. Furthermore, based on the functional properties of PLDs, notably as activators of the inflammatory response and stimulants of the production of different cytokines and modulators of cells or cell fragments, such as endothelial cells, fibroblasts, platelets, erythrocytes, leukocytes, etc. [2,8], these PLDs are potential targets in the design of experimental protocols to obtain new drugs that can inhibit inflammatory processes, modulate the immune system, act on vascular structures or blood cells, or be used as a specific treatment for systemic loxoscelism. The interactions of PLDs with different cell types make these molecules useful in the discovery of putative cellular receptors. In addition, the interaction profile between PLDs and cellular phospholipids may enable novel cell biology discoveries concerning intracellular signal cascades generated after binding with different cells, providing useful insights in the intracellular and molecular pathways that could be targets for putative inhibitors. The high biochemical and biological functionality of these enzymes confer enormous potential to the PLDs of *Loxosceles* spider venoms for use in future discoveries based on these molecules will be realities.

## Figures and Tables

**Table 1:** Main retrospective studies and case reports of intravascular hemolysis and hemolytic anemia in systemic loxoscelism.

Most Common Systemic Signs and Symptoms Related to Hemolysis	Most Common Laboratory Findings Related to Hemolysis	Total Number of Cases	Ref.
Jaundice; dark urine; malaise; fever	Hemoglobinuria; low hematocrit; direct Coombs positive	2	[24]
Jaundice; fever; signs of hemolytic anemia	-	6	[25]
Jaundice; dark/red urine; fever; pallor; diffuse rash; fatigue	Anemia; positive DAT ^1^	6	[23]
Jaundice; hemolytic anemia	Anemia; hematuria	26	[26]
Dark urine; hemolytic anemia; fever; rash	Low hemoglobin; direct Coombs positive; increased LDH ^2^ and indirect bilirubin	1	[27]
Hemolytic anemia	Low hemoglobin; increased LDH ^2^ and total bilirubin; positive DAT ^1^; hematuria	373	[28]
Jaundice; hemolytic anemia; fever; rash	Low hemoglobin; positive DAT ^1^	9	[29]
Malaise; fever; exanthem; hemolysis	Low hemoglobin; low hematocrit	97	[19]
Low oxygen saturation; dark urine; abdominal pain; nausea; vomiting; intermittent fever; profound intravascular hemolysis	Low hemoglobin; abnormal coagulation profile; hemoglobinuria; myoglobinuria; increased whole blood lactate; direct Coombs positive test	1	[31]
Fatigue; dyspnea; fever; hemolysis	Low hemoglobin; increased serum lactate; increased creatinine, LDH ^2^, C-reactive protein and total bilirubin; positive DAT ^1^	1	[32]
Dark urine; fever; body aches; rash; nausea; hemolysis	Low hemoglobin; increased bilirubin; direct Coombs positive	1	[33]
Red urine; fever; bilateral scleral icterus.	Low hemoglobin; low hematocrit; increased bilirubin, LDH ^2^ and C-reactive protein; positive DAT ^1^; reticulocytosis	2	[30]

^1^ DAT: Direct agglutination test. ^2^ LDH: Lactate dehydrogenase.

**Table 2:** Main retrospective studies and case reports of acute renal failure (ARF) in systemic loxoscelism.

Most Common Systemic Signs and Symptoms Related to ARF	Most Common Laboratory Findings Related to ARF	Total Number of Cases	Ref.
Oliguria; vomiting; jaundice; fever; severe hemolysis and rhabdomyolysis	Increased plasma creatinine and blood urea	2	[44]
Hypotension; hemolytic anemia	Increased potassium levels	6	[25]
Rhabdomyolysis	Increased serum creatine phosphokinase; hematuria	26	[26]
Oliguria; hypotension; dark-red-colored urine; vomiting; fatigue; fever	Proteinuria; hematuria; pyuria; increased creatinine and blood urea levels; heterogeneous enhancement pattern of kidneys found in tomography of abdomen and pelvis	1	[45]
Oliguria; dark urine; periumbilical pain; vomiting; headache; malaise	Increased serum creatine kinase and urea; kidney biopsy with acute tubular damage; pigment casts in some tubules; interstitial edema	1	[46]
Nausea; vomiting; malaise	Increased serum creatinine, urea, and potassium.	45	[47]
Hypotension, fever; tachycardia	Increased serum creatinine	9	[29]

**Table 3:** Main retrospective studies and case reports of alterations of hemostasis and blood coagulation in systemic loxoscelism.

Most Common Findings Related Alterations of Hemostasis and Blood Coagulation	Total Number of Cases	Ref.
Increased plasma fibrinogen, D-dimer, and CRP ^1^ levels; thrombocytopenia; leukocytosis; neutrophilia	81	[22]
Increased fibrinogen, D-dimer, and CRP ^1^ levels; severe thrombocytopenia; reduced APTT ^2^, presence of platelet antibodies in plasma; petechial and diffuse morbilliform rash on the limbs, trunk, and face	1	[52]

^1^ CRP: C-reactive protein. ^2^ APTT: Activated partial thromboplastin time.

**Table 4:** Main retrospective studies and case reports of neurologic and cardiac disturbances after *Loxosceles* bite.

	Findings Related to Neurologic and Cardiac Disturbances	Total Number of Cases	Ref.
Neurologic	Signs of optic neuropathy, characterized by a left relative afferent papillary defect, bilateral optic nerve pallor, decreased foveal sensitivity in the left eye, and bilateral visual compound defects	1	[67]
Cardiac	Tachycardia; hypotension	9	[29]
	Exertional dyspnea; lower limb edema; tachycardia and diastolic heart failure due to a systemic lupus erythematosus flare triggered by the bite	1	[68]
	Tachycardia; hypotension; dyspnea; mild pulmonary edema; increased B-type natriuretic peptide, troponin-I, and creatine kinase-muscle/brain levels; myocarditis involving the left ventricular apex and basal portion of the heart, confirmed by cardiac magnetic resonance imaging	1	[69]

## Data Availability

Not applicable.
